# An Aptamer-Based Biosensor for Direct, Label-Free Detection of Melamine in Raw Milk

**DOI:** 10.3390/s18103227

**Published:** 2018-09-25

**Authors:** Naoto Kaneko, Katsunori Horii, Joe Akitomi, Shintaro Kato, Ikuo Shiratori, Iwao Waga

**Affiliations:** NEC Solution Innovators, Innovation Laboratory, 1-18-7, Shinkiba, Koto-ku, Tokyo 136-8627, Japan; nao-kaneko@ys.jp.nec.com (N.K.); jou-akitomi@vt.jp.nec.com (J.A.); shi-kato@ts.jp.nec.com (S.K.); iku-shiratori@xc.jp.nec.com (I.S.); iwa-waga@wm.jp.nec.com (I.W.)

**Keywords:** aptamer, DNAzyme, biosensor, label-free detection, melamine, sample preparation

## Abstract

Melamine, a nitrogen-rich compound, has been used as a food and milk additive to falsely increase the protein content. However, melamine is toxic, and high melamine levels in food or in milk can cause kidney and urinary problems, or even death. Hence, the detection of melamine in food and milk is desirable, for which numerous detection methods have been developed. Several methods have successfully detected melamine in raw milk; however, they require a sample preparation before the analyses. This study aimed to develop an aptamer-DNAzyme conjugated biosensor for label-free detection of melamine, in raw milk, without any sample preparation. An aptamer-DNAzyme conjugated biosensor was developed via screening using microarray analysis to identify the candidate aptamers followed by an optimization, to reduce the background noise and improve the aptamer properties, thereby, enhancing the signal-to-noise (S/N) ratio of the screened biosensor. The developed biosensor was evaluated via colorimetric detection and tested with raw milk without any sample preparation, using *N*-methylmesoporphyrin IX for fluorescence detection. The biosensor displayed significantly higher signal intensity at 2 mM melamine (S/N ratio, 20.2), which was sufficient to detect melamine at high concentrations, in raw milk.

## 1. Introduction

Melamine is a nitrogen-rich compound widely used as a manufacturing material. Unfortunately, melamine contamination in food has been a persistent issue owing to its toxicity; the most severe of which is the large-scale adulteration of milk and pet food, with melamine, to falsely increase the protein content. Melamine at high concentrations can cause kidney and urinary problems or even death [1,2,3]. Hence, it is desirable to detect melamine contamination in food to prevent its ingestion, for which numerous methods have been developed.

Accurate and highly sensitive methods involving liquid chromatography/mass spectrometry (LC/MS) [4], gas chromatography/mass spectrometry (GC/MS) [5], and high-performance liquid chromatography (HPLC) [6] have been developed.; However, these methods are costly and require skill and complex, time-consuming, preprocessing of samples. Moreover, food samples must be transported to the laboratory for analysis. To circumvent these problems, many sensor-type on-site detection methods have been developed, and melamine sensors based on gold nanoparticles (AuNPs) showed notable success for the rapid, on-site detection of melamine in milk with easy sample preparation [7,8]. However, these methods still require sample preparation for raw milk, despite being easier than the methods involving instrumental analysis. For practical analysis of melamine, detection, without any samples preparation is still required.

The peroxidase-mimicking DNAzyme is a guanine-rich oligonucleotide that forms a G-quadruplex structure with a redox activity toward hemin and peroxide [9,10]. Aptamers generated via systematic evolution of ligands, by an exponential enrichment (SELEX) [11,12], were conjugated with the DNAzyme to form a label-free biosensor [13]. The aptamer-DNAzyme conjugated biosensor requires block sequences of appropriate length, for both the aptamer and the DNAzyme, and for the internal loop sequences, to couple the aptamer and DNAzyme to the block sequences. Owing to the numerous combinations of the sequence lengths, redox activity microarray technology has been used to screen biosensors [14,15]. Moreover, the DNAzyme activity increases the fluorescence signal intensity of *N*-methylmesoporphyrin IX (NMM) [16,17]. Raw milk contains several compounds that inhibit redox activity. However, using NMM instead of hemin allows for the detection of melamine in raw milk, without any sample preparation.

This study aimed to develop an aptamer-DNAzyme conjugated biosensor, for melamine detection in raw milk. The process included a microarray-based screening and optimization of the screened biosensor, via chemiluminescence detection. Thereafter, the optimized biosensor was subjected to colorimetric analysis of the melamine contamination, in the buffer samples. Finally, the sensor was evaluated in raw milk, without any sample preparation, via fluorescence detection of the NMM. The aptamer-DNAzyme conjugated biosensor was thus able to successfully detect melamine at high concentrations in raw milk.

## 2. Materials and Methods

### 2.1. Chemicals and Reagents

Porcine hemin and *N*-methylmesoporphyrin IX (NMM) were purchased from Sigma-Aldrich (St. Louis, MO, USA). Ferrocene methyl alcohol (FMA), melamine monomer, and 2,2′-azino-bis(3-ethylbenzothiazoline-6-sulfonic acid ammonium salt) (ABTS) were purchased from Tokyo Chemical Industry (Tokyo, Japan). Hydrogen peroxide (30%) was purchased from Wako Pure Chemical Industries (Osaka, Japan). DNA oligonucleotides were synthesized by Integrated DNA Technologies (Coralville, IA, USA). UltraPure DNase/RNase-free distilled water (Invitrogen, Carlsbad, CA, USA) was used for all experiments. L-012 was purchased from Wako Chemicals USA, Inc. (Richmond, VA, USA). All other chemicals and reagents were of molecular biology grade. Raw milk (Meiji, Japan) was purchased from the supermarket.

### 2.2. Sequence Design for Screening an Aptamer-DNAzyme Conjugated Biosensor

**Figure 1:**
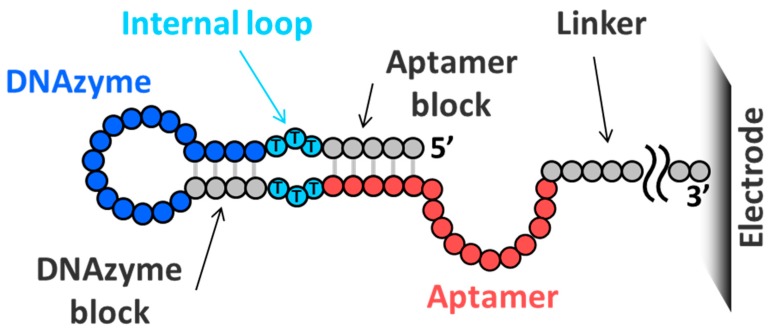
A schematic representation of the design of an aptamer-DNAzyme conjugated biosensor for microarray screening. The designed biosensor comprises an aptamer region (red), a DNAzyme region (blue), an internal loop region (cyan), two block sequences (gray) for the aptamer and the DNAzyme, and a linker region, immobilized on the microarray electrode.

Twelve melamine aptamers modified from the original poly-T melamine aptamer [18], and a designed peroxidase-mimicking DNAzyme [19], were used for screening (Table 1). The original poly-T aptamer dimerized and bound to melamine. However, the modified melamine aptamer comprises a stem-loop structure that bound the melamine, without any dimer formation. The blocking sequences for the aptamer and the DNAzyme sequence were complementary and formed the base-stacking structures. The internal loop sequences were poly-T, as was the linker sequence from the electrode microarray. A schematic representation of the design of the aptamer is shown in Figure 1. The lengths of the aptamer/DNAzyme blocking sequences were 0, 3, 4, 5, and 6, and the lengths of the internal loop sequences were 0, 1, 3, and 5. All sequences were designed as 80-mers with changes in the length of the linker sequence. For example, if the aptamer and DNAzyme blocking sequences, internal loop sequences, and the melamine aptamer were 5 nt, 5 nt, 3 nt, and Mel01 (a 13-mer), respectively, then the length of the linker sequence was 35 = 80 − 13 − 5 − 5 − 3 − 3 − 16 (length of the DNAzyme). The distance between the DNAzyme and the electrode exerts a strong effect on redox activity [19]; hence, the DNAzyme should have been located a similar distance from the electrode, across all sequences. The 1200 unique sequences, 1180 for sensor screening, and 20 as control sequences, were randomly located on the 12K ElectraSense microarray chip purchased from Recenttec K.K. (Tokyo, Japan), with ten replicates. A 6-mer DNAzyme blocking sequence for Mel01 was excluded owing to the limited number of spots on the microarray.

There were twelve modified melamine aptamers and peroxidase-mimicking DNAzyme. Twelve modified melamine aptamers were designed by including a stem region and changing the length of the poly-T region. The original aptamer contained a 31-mer poly-T sequence [18]. The biosensor was designed from these modified melamine aptamers and the DNAzyme, and was evaluated with microarrays.

### 2.3. Electrochemical Detection of DNAzyme Activity via Microarray Analysis for Screening Biosensor Candidates

The designed oligonucleotides were directly synthesized on the microarray (purchased from Recenttec K.K., Tokyo, Japan). The microarray chip was immersed in 200 μL of DNAzyme buffer (50 mM Tris-HCl, 20 mM KCl, and 0.05% [*w*/*v*] Triton X-100, pH 7.4) and incubated for 10 min, at 75 °C. The chip was then gradually cooled to the room temperature (25 °C), for more than 1 h, to allow the probes to fold into their correct conformations. The buffer was replaced with hemin solution (5 μM hemin in the DNAzyme buffer, containing 0.1% [*v*/*v*] DMSO) without the melamine and incubated for 30 min, at room temperature, with a gentle agitation in the dark. The hemin solution was replaced with the substrate solution (2 mM H_2_O_2_ and 0.1 mg/mL FMA in hemin solution) without the melamine and mixed gently via repeated pipetting before the microarrays were scanned. The chip was immediately washed, thrice, with DNAzyme buffer, to eliminate the substrate solution after the scanning of the microarray chip. Signal intensity (redox activity of hemin and substrate) was measured using an ElectraSense Reader (CombiMatrix, Irvine, CA, USA) [20]. The same procedure was repeated with the hemin and the substrate solutions, containing 10 mM melamine to measure the signal intensity, in the presence of melamine. The signal-to-noise (S/N) ratio was calculated from the ratio between the median signal intensities of the ten replicates of no melamine, and those containing 10 mM melamine.

### 2.4. Optimization of the Aptamer-DNAzyme Conjugated Biosensor

The screened aptamer-DNAzyme conjugated biosensor was optimized in three steps—reduction of the background noise, adjustment of the aptamer stability to attain the appropriate conformation to bind the melamine, and the extension of the poly-T melamine aptamer to improve the affinity. To reduce background noise, the block sequence for the DNAzyme was extended. After sufficient reduction of background noise, the stem region of the aptamer was extended to enhance stability. Chemiluminescence detection was performed to evaluate the optimization steps.

### 2.5. Chemiluminescence Detection

A DNA solution (20 pmol of the biosensor in 25 μL of 2× DNAzyme buffer [100 mM Tris-HCl and 0.1% [*w*/*v*] Triton X-100, pH 7.4]) was denatured for 5 min, at 95 °C. The solution was then gradually cooled to the room temperature to allow for an attainment of the appropriate conformation. Hemin in DMSO and KCl (final concentrations of 200 nM and 20 mM, respectively) were added, and the volume was adjusted to 20 μL, with a DNAzyme buffer. A sample of 25 μL of the solution, with or without melamine, was added and incubated for 15 min at the room temperature. The substrate solution, which includes 2.5 μL of L-012, as a luminol derivative (final concentration of 25 μM), and 2.5 μL of H_2_O_2_ (final concentration of 25 μM), was injected into the DNAzyme solution. The chemiluminescence intensity was measured immediately, using a TECAN Infinite 200 reader (TECAN Japan, Kawasaki, Japan). A hemin solution (200 nM hemin in DNAzyme buffer with no DNAzyme oligonucleotide) was used as a negative control.

### 2.6. Colorimetric Detection

A DNA solution (100 pmol of the biosensor in 50 μL of 2× DNAzyme buffer [100 mM Tris-HCl and 0.1% [*w*/*v*] Triton X-100, pH 7.4]) was denatured for 5 min, at 95 °C. The solution was then cooled gradually to the room temperature, to allow for the attainment of the appropriate conformation of the sensor. Hemin in DMSO and KCl (final concentrations of 3 μM and 20 mM, respectively) were added. Sample solutions were spiked with each diluted concentration of melamine and incubated for 15 min, at the room temperature. Thereafter, ABTS (final concentration, 1 mM) and H_2_O_2_ were added to the DNA solution. The total volume was 100 μL. The absorbance of each sample was measured at 415 nm, by a TECAN Infinite 200 reader.

### 2.7. Fluorescence Detection

A DNA solution (20 pmol of the biosensor in 25 μL of 2× DNAzyme buffer [100 mM Tris-HCl, 100 mM EDTA and 0.1% [*w*/*v*] Triton X-100, pH 7.4]) was denatured for 5 min, at 95 °C. The solution was then gradually cooled to the room temperature, to allow for the attainment of the appropriate conformation of the sensor. NMM and KCl (final concentrations of 200 nM and 20 mM, respectively) were added. Thereafter, 25-μL samples of raw milk were spiked with each dilution of melamine, and incubated for 10 min, at room temperature. The fluorescence intensity was measured using a TECAN Infinite M1000 Pro (TECAN, Zürich, Switzerland), with an extraction wavelength of 399 nm and an emission wavelength of 605 nm.

## 3. Results and Discussion

The redox activity of the DNAzyme of the biosensor was obtained with an ElectraSense Reader as the signal intensity, with or without target molecules. The S/N ratio of the microarray screening is shown in the heatmap (Supplementary Figure S1). This heat map shows the relation between the modified melamine aptamers (the rows) and the length of aptamer/DNAzyme block sequences, and the internal loop sequences (the columns). The top-ranked sequence was tGGGTGGGAGGGTCGGG**ccc**tCGCTTTTTTTTTTTTGCG (Sensor 1), and its S/N ratio was 2.1 (= 5510/2564). This screened biosensor had no block sequence for the aptamer region, had three bases for the DNAzyme block sequence (bold **ccc**) and one base for the internal loop sequences (underlined t). As the subsequent (**ccc**tC), which included the aptamer stem region, could stack directly with a part of the DNAzyme (GGGAG), the aptamer region did not require a block sequence, in this case. Most of the sequences containing no aptamer block sequence showed better S/N ratios (Supplementary Figure S2). This specific case was different from that of the streptavidin biosensor, the AMP biosensor [13], and the patulin biosensor [14].

**Figure 2:**
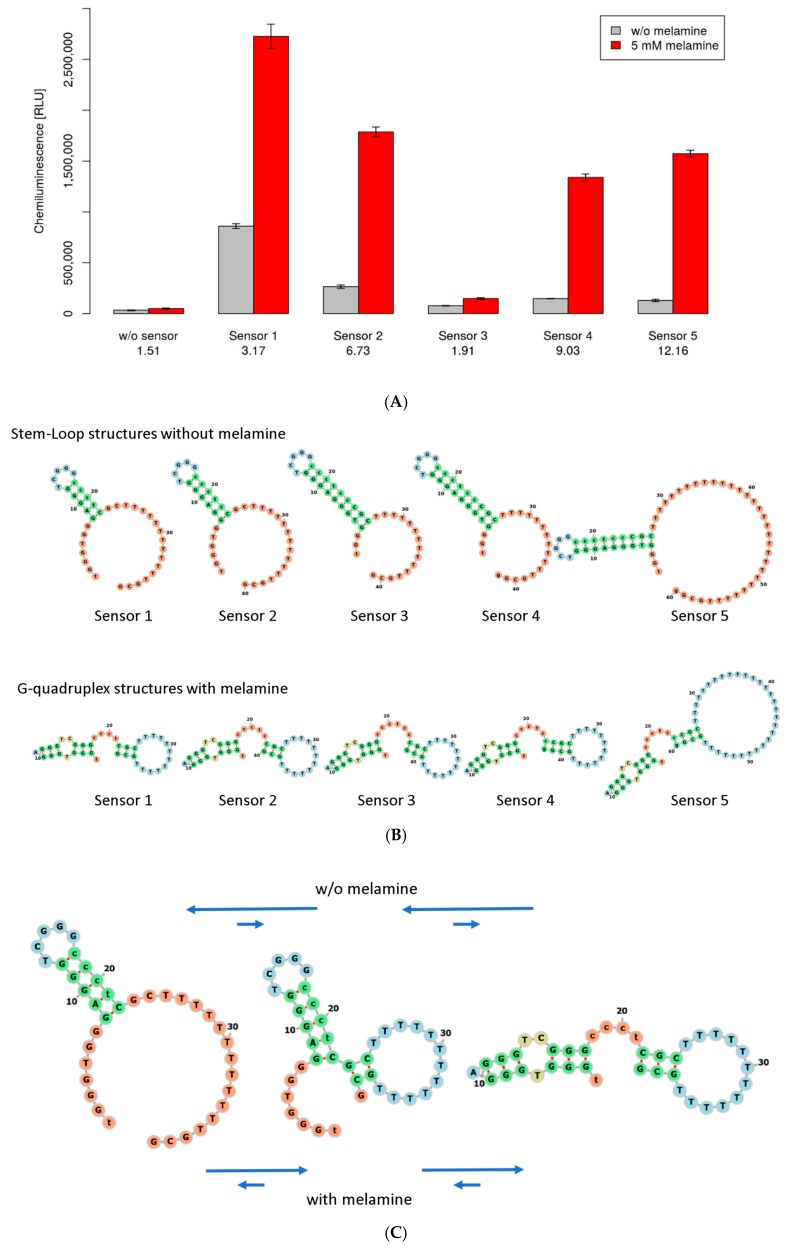
(**A**) The signal intensity for each step. The small vertical line in the bar chart represents the standard error (N = 3). Gray bars and red bars show the signal intensity without melamine and with 5 mM melamine, respectively. The signal-to-noise ratio for each step is shown at the bottom of the figure. (**B**) The estimated secondary structure of the biosensors at each optimization step. The top row and bottom row show the secondary structure without the G-quadruplex and with the G-quadruplex, respectively. The secondary structures were estimated with RNAfold [21] and generated with Forna [22]. (**C**) The change in the folding Sensor 1. The arrows show the equilibrium both without melamine and with melamine.

During optimization, the screened biosensor (Sensor 1) was modified four times to produce Sensor 2 to Sensor 5, sequentially. The sequences of each biosensor are shown in Table 2. The chemiluminescence of solutions containing no melamine and that of solutions containing 5 mM melamine are shown in Figure 2A. The numbers at the bottom of the figure represent the S/N ratio. Sensor 1 showed a high background signal; therefore, the block sequence for the DNAzyme was extended to one base (Sensor 2), and then to two bases (Sensor 3). Sensor 3 showed a lower background signal; however, it did not display the chemiluminescence signal of melamine because the stem region of the aptamer was less stable than the stem of a DNAzyme, and the DNAzyme did not change the structure of the G-quadruplex. Extension of the stem region, by one base, reverted to the high signal intensity of the sensor (Sensor 4). The poly-T was extended to 60-mer total (Sensor 5), and then the sensor showed the best S/N ratio, which was improved from 3.2 to 12.2 (improved by 3.8×). The combination of the screening and the design optimization of the DNA sequence was a useful approach for developing biosensors. The secondary structure of each sequence was estimated using the ViennaRNA package [21], and Figure 2B was generated using Forna [22]. The top and bottom rows of secondary structures show the stem-loop and G-quadruplex structures, respectively. The structures of the stem-loop and the G-quadruplex are very different; hence, it was possible to form an intermediate structure. Figure 2C shows the change from a stem-loop to a G-quadruplex structure with a suboptimal intermediate structure. The arrows show the equilibrium between structures with or without melamine.

Sensor 1 was identified via a microarray screening. Sensors 2 and 3 were designed to reduce background noise by extending the DNAzyme block sequence. Sensor 4 was designed to improve the stability of the aptamer region by extending its stem region. Sensor 5 included the poly-T sequence, which was extended to form a 60-mer, in total. The changes from Sensor 1 are shown in boldface. The signal-to-noise ratio for each step is also shown.

**Figure 3:**
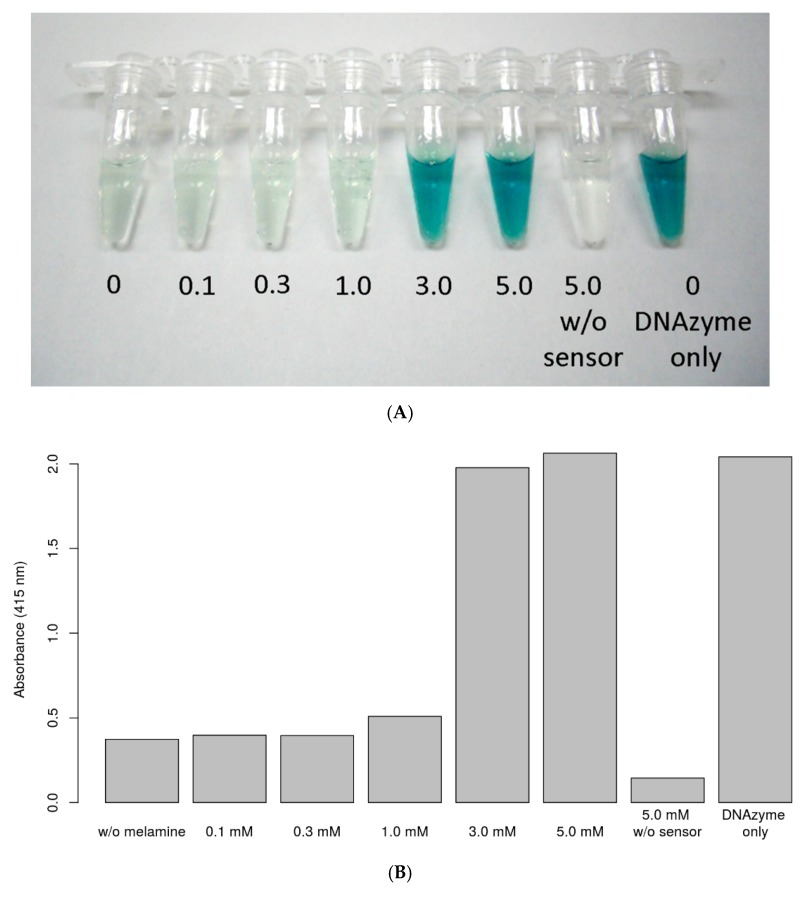
(**A**) Colorimetric detection of different concentrations of melamine in the buffer. The melamine concentrations from left to right were 0.0 mM, 0.1 mM, 0.3 mM, 1.0 mM, 3.0 mM, 5.0 mM, 5.0 mM (w/o sensor), and 0 mM (DNAzyme only). (**B**) Absorbance detected at different melamine concentrations. The absorbance was measured at 415 nm, for 2,2′-azino-bis(3-ethylbenzothiazoline-6-sulfonic acid ammonium salt).

The optimized biosensor was subjected to colorimetric detection, and Figure 3A shows the color change in relation to the melamine concentration in the buffer. The color difference can be recognized by the naked eye for 3 mM melamine. The color change in the absorbance is shown in Figure 3B.

Inhibition of redox activity in raw milk was assessed via chemiluminescence detection. Redox activity displayed a weaker signal at 0.01% than at 0% milk. Redox activity in 1% and 10% milk was quite low (Supplementary Figure S3). Therefore, the melamine cannot be detected in raw milk on the basis of redox activity; hence, other detection methods are warranted.

**Figure 4:**
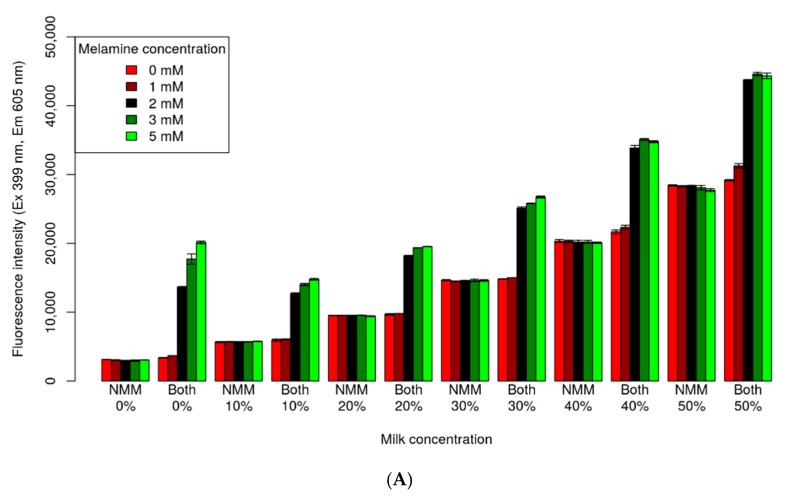

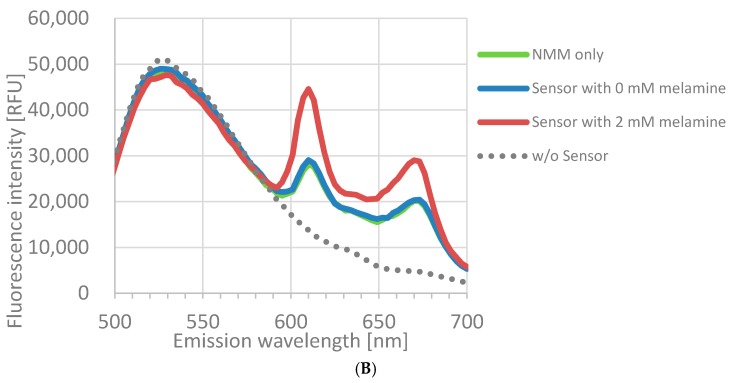
(**A**) Fluorescence intensity at different concentrations of milk and melamine. The *N*-methylmesoporphyrin IX (NMM) and both labels at the bottom of the bar chart represent NMM alone and NMM with the biosensor, respectively. The milk concentration is also shown at the bottom of the bar plot and ranged from 0% to 50%. The color represents melamine concentrations from 0 mM to 5 mM. The excitation and emission wavelengths for excitation and emission were set at 399 nm and 605 nm, respectively. (**B**) Fluorescence spectra of NMM alone and of both NMM and melamine. The milk concentration was 50%, and the excitation wavelength was set to 399 nm. The green, blue, and red lines represent NMM alone with 0 nM melamine, NMM plus a biosensor with 0 mM melamine, and NMM plus biosensor at 2 mM melamine, respectively. The dotted line represents the fluorescence intensity of milk.

Fluorescence detection was performed instead of analysis of redox activity, to evaluate the biosensor for use in raw milk, without any sample preparation. Figure 4A shows the fluorescence intensity with respect to milk and melamine concentrations. Addition of NMM alone did not affect melamine concentration. However, the addition of both NMM and the sensor significantly increased the signal intensity with 2, 3, and 5 mM melamine, compared to the control. The gradient of the signal intensity was related to milk concentration. However, the signal of NMM alone showed the same intensity as that of 0 mM melamine, across all concentrations. Thus, the signal intensity of NMM alone could serve as a reference for the background intensity. Figure 4B showed the fluorescence spectra of a melamine biosensor of 50% milk concentration, with NMM alone and 0 mM melamine, of both, the NMM and the biosensor, for 0 mM and 2 mM melamine, and for raw milk alone. Fluorescence intensity peaked at an emission wavelength of 605 nm, wherein the S/N ratio between 0 mM and 2 mM was probably 1.5, and NMM alone showed the same spectrum with 0 mM of melamine (Figure 4B). Upon normalization of the signal intensity to the background intensity of NMM, it only yielded a normalized S/N ratio of 20.2. Increased melamine content was greater than 500 ppm (classified as high-melamine content [1]), which was equal to 3.96 mM. This aptamer biosensor showed sufficient ability to detect melamine in high-melamine milk without the need for sample preparation.

The aptamer-DNAzyme conjugated biosensor for melamine detection was developed using a combination of screening and optimization procedures. Screening with microarray technology proved useful in identifying the approximate length of the block and the internal loop sequences, from among numerous sequence designs. Optimization helped reduce the background noise and improve aptamer properties, and the resulting S/N ratio was greatly improved by the screened biosensor. The developed biosensor was evaluated via colorimetric detection, and the color gradients were related to the concentration of melamine, in the buffer. To address its practical application, the biosensor was also assessed with raw milk without any sample preparation, by using NMM for fluorescence detection. The biosensor showed significantly higher signal intensity at 2 mM melamine, which was sufficient to detect melamine at high concentrations, in milk.

Such convenient fluorescence detectors are currently in use. Therefore, biosensors with a portable detector using the same wavelength as that described in our study, have the potential, among those available globally, to provide real on-site detection of melamine. This approach is cost-effective compared to the use of AuNP-based biosensors. In general, oligonucleotides and NMM are not toxic; hence, the biosensor can be assessed safely. Owing to these advantages, biosensors could have potential practical applications.

## Figures and Tables

**Table 1:** Melamine aptamers modified from the original poly-T melamine aptamer and a designed peroxidase-mimicking DNAzyme used for screening.

Name	Function	Length	Length of Poly-T	Sequence
Mel01	Melamine Aptamer	13	7	CGCTTTTTTTGCG
Mel02	Melamine Aptamer	14	8	CGCTTTTTTTTGCG
Mel03	Melamine Aptamer	15	9	CGCTTTTTTTTTGCG
Mel04	Melamine Aptamer	16	10	CGCTTTTTTTTTTGCG
Mel05	Melamine Aptamer	17	11	CGCTTTTTTTTTTTGCG
Mel06	Melamine Aptamer	18	12	CGCTTTTTTTTTTTTGCG
Mel07	Melamine Aptamer	17	7	GCGCGTTTTTTTCGCGC
Mel08	Melamine Aptamer	18	8	GCGCGTTTTTTTTCGCGC
Mel09	Melamine Aptamer	19	9	GCGCGTTTTTTTTTCGCGC
Mel10	Melamine Aptamer	20	10	GCGCGTTTTTTTTTTCGCGC
Mel11	Melamine Aptamer	21	11	GCGCGTTTTTTTTTTTCGCGC
Mel12	Melamine Aptamer	22	12	GCGCGTTTTTTTTTTTTCGCGC
DNAzyme	DNAzyme	16	-	GGGTGGGAGGGTCGGG

**Table 2:** Sequence information for each optimization step.

Name	S/N Ratio	Length	Sequence
Sensor 1	3.2	39	TGGGTGGGAGGGTCGGGCCCTCGCTTTTTTTTTTTTGCG
Sensor 2	6.7	40	TGGGTGGGAGGGTCGGGCCCT**C**CGCTTTTTTTTTTTTGCG
Sensor 3	1.9	41	TGGGTGGGAGGGTCGGGCCCT**CC**CGCTTTTTTTTTTTTGCG
Sensor 4	9	42	TGGGTGGGAGGGTCGGGCCCT**CC**CGCTTTTTTTTTTTTGCG**G**
Sensor 5	12.2	60	TGGGTGGGAGGGTCGGGCCCT**CC**CGCTTTTTTTTTTTT**TTTTTTTTTTTTTTTTTT**GCG**G**

## Supplementary Materials

The following are available online at http://www.mdpi.com/1424-8220/18/10/3227/s1, Figure S1: S/N ratios for all designed sequences, Figure S2: The relation of signal intensity between no-melamine and 10 mM melamine, Figure S3: The relation between chemiluminescence intensity and the concentration of milk.
