# Supplementary material for: An Aptamer-Based Biosensor for Direct, Label-Free Detection of Melamine in Raw Milk
Source: Sensors (Basel). 2018 Sep 25;18(10):3227. doi: 10.3390/s18103227 (PMC6210019; doi:10.3390/s18103227)
Supplement: Supplementary file 1 [file sensors-18-03227-s001.pdf]

sensors

MDPI

Supplementary Information for The Aptamer-Based Biosensor of Label-Free, Direct Detection of Melamine in Raw Milk

Naoto Kaneko, Katsunori Horii, Joe Akitomi, Shintaro Kato, Ikuo Shiratori, and Iwao Waga

NEC Solution Innovators, Innovation Laboratory, 1-18-7, Shinkiba, Koto-Ku, Tokyo 136-8627, Japan

Supplementary figure 1
This heat map shows the S/N ratios for all designed melamine biosensors. Row names are related to the name of the modified melamine aptamers and the columns are related to the length of aptamer/DNAzyme block sequences and the internal loop sequences. For example, the combination of A0, D3, and I1, mean no block sequence for aptamer, 3-mer for DNAzyme block sequence, and one base for the internal loop. The highest S/N ratio is 2.1 with A0, D3, and I1. The color reference is shown on the right of the heatmap.

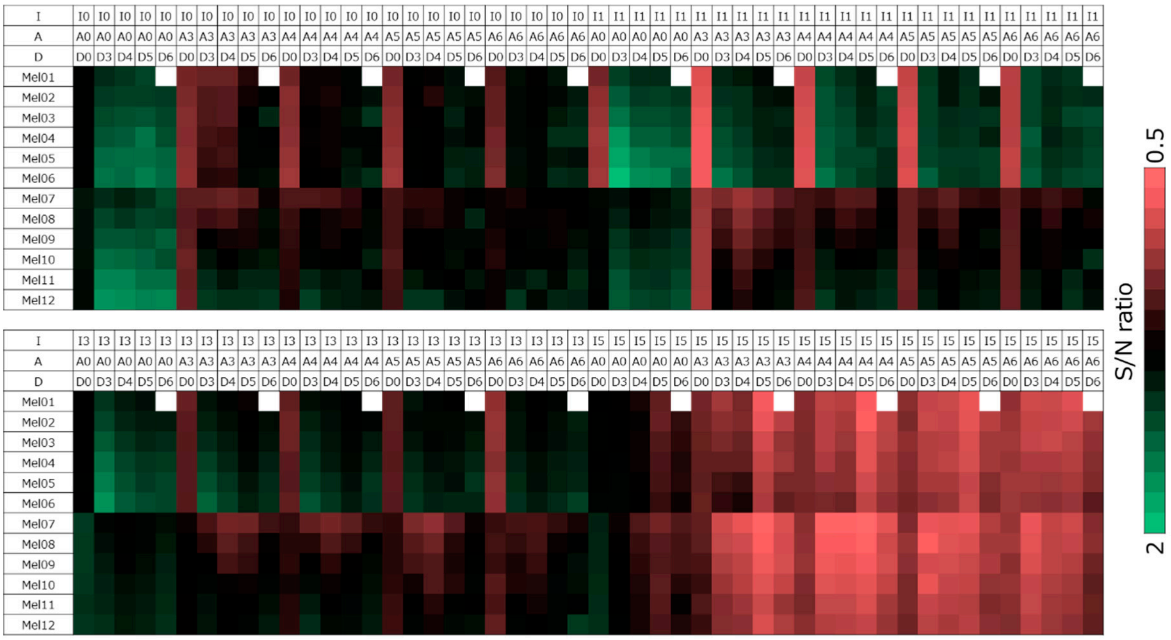

Figure S1. S/N ratios for all designed sequences.

Supplementary Figure 2

Color represents the length of the block sequences for aptamer. A0, A3, A4, A5, and A6 mean the lengths of aptamer block sequences for no block sequence, 3, 4, 5, and 6, respectively. The dotted diagonal line represents that the S/N ratio is equal to 1.

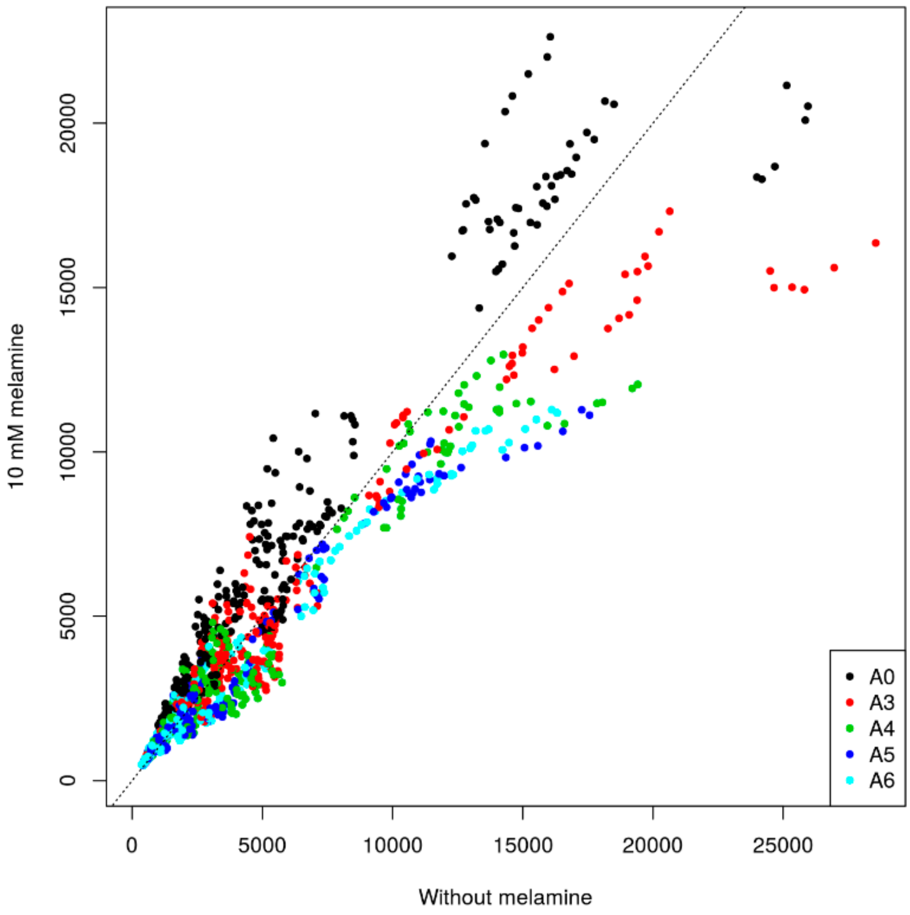

Figure S2. The relation of signal intensity between no-melamine and 10 mM melamine.

Supplementary Figure 3

The protocol of experimental analysis is shown as follows. DNA solution (20 pmol of DNAzyme in 25 μL of 2x DNAzyme buffer [100 mM Tris-HCl and 0.1% [w/v] Triton X-100, pH 7.4]) was denatured for 5 min, at 95 °C. The solution was then cooled down, slowly, to the room temperature, for proper structure of the biosensor. Hemin in DMSO and KCl (final concentration of 200 nM and 20 mM, respectively) were added, and filled up to 20 μL with the DNAzyme buffer. Raw milk of 25 μL was diluted with distilled water (final concentration: 0, 0.0001, 0.001, 0.01, 0.1, 1, 10% raw milk), it was added and incubated for 15 min, at room temperature. Next, 2.5 μL of L-012 as a luminol derivative (final concentration of 25 μM) and 2.5 μL of H_2O_2 (final concentration of 25 μM) were injected into the DNA solution. The chemiluminescent reaction was measured immediately by the TECAN infinite 200 reader from the TECAN.

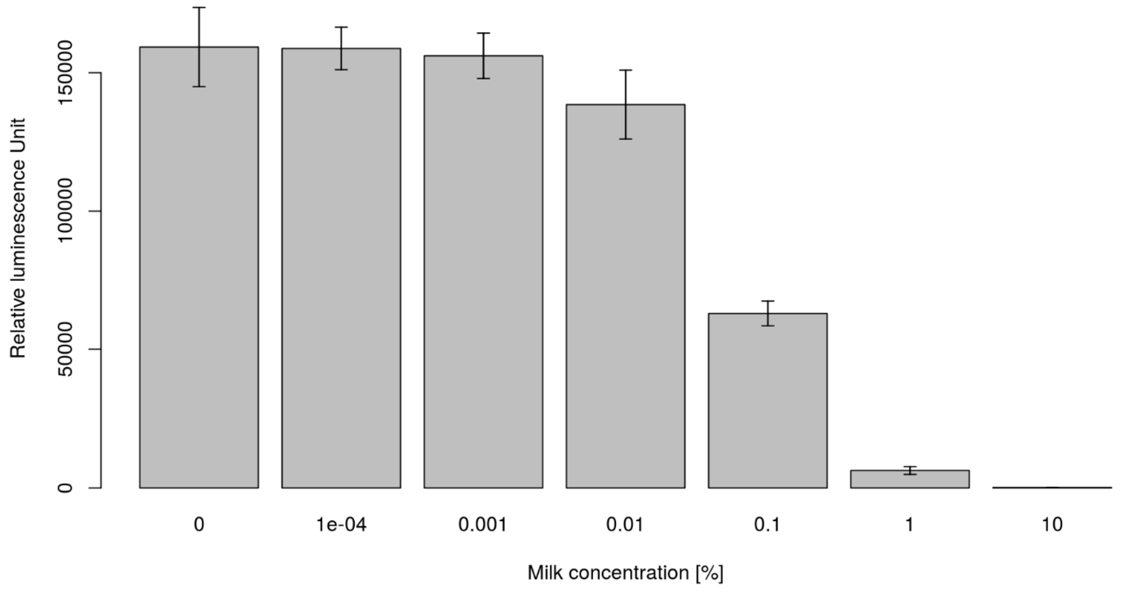

Figure S3. The relation between the chemiluminescence intensity and the concentration of milk.
